# Editorial Chit-Chat

**Published:** 1875-04

**Authors:** 


### Editorial Chit-Chat.

---

EX-MAYOR CALDWELL is fishing for alligators on the St. John's river in Florida. We hear he takes them on a fly.

---

PILLS.—Mr. Holloway, vender of pills and ointment, has given one million dollars to establish a college for women near Egham.

---

BEHIND TIME.—We are late with this issue of our magazine, in consequence of procuring the new materials with which it is printed.

---

RELIGIOUS INSANITY.—Several cases of religious insanity have resulted from the revival meetings of Messrs. Moody and Sankey, in England.

---

JULY NUMBER.—Should it be a few days late, also, conclude that the editor has "gone a fishing," and will be able to give you a better number, by reason of his much needed rest.

---

LIVINGSTONE'S HUMERUS.—The Chicago Medical College has obtained a plaster cast of the large bone of the arm of the great traveler, showing the famous false joint caused by the jaws of the lion.

---

CORK IN CALIFORNIA.—California, it is said, is succeeding in cork growing. Artificial forests of cork trees have been planted which have already grown sufficiently to produce a bark from which a cork can be cut, an inch in diameter.

---

DISSECTING ROOM SCENE.—The janitor of an Indianapolis, Ind., Medical College was deeply affected on recognizing his mother-in-law on the dissecting table. His grief was the more piquant from the fact that he had himself carried the stolen corpse up three flights of stairs.

---

A DIFFICULT ORDER.—One of the proprietors of a merchant-tailoring establishment in this city had occasion recently, to make an order for two of the large irons used in pressing clothes, and technically called a "goose." He was much perplexed how to write the plural of the object desired. First he wrote: "Please send me two "geese"—this jingled harshly upon his ear; then he wrote "gooses," that seemed to satisfy him still less, so that in sheer despair he finally settled upon the following order: "Please send me a medium sized goose,— d——n it, send *two* of them," and so extricated himself from the dilemma.

DRUNKEN BABIES.—An eminent English lecturer, in an address to an audience of women recently, in London, asserted that a majority of the babies of that city "are never sober from their birth until they are weaned." This, because of the mothers being addicted to milk punch, brandy toddies, &c.

---

PHOTOGRAPH THEIR HANDS.—An ingenious gentleman, probably a pupil of some gypsy, has discovered that the lines upon the palm of the hand are never alike in any two persons. He recommends that the palms of the hands of criminals be photographed, and preserved as a means of recognizing them when occasion requires.

---

A REASON FOR IT.—Our Nebraska patient announced, early in the winter, the cause of the dearth of snow to be that, the grasshoppers had eaten it, attributing to those much abused insects about all the misfortunes that usually befall man. Recent developments, in this latitude, have convinced us that the *hymenoptera* have at last become surfeited, and that we are now reaping the benefit of their condition.

---

CO-EDUCATON OF THE SEXES.—The Michigan University, where both sexes are admitted, revealed the following facts at its last commencement: The whole class numbered three hundred and sixty, and of these, fifty were women. The women carried off a large share of the honors, and it is admitted that they generally stand higher in their studies than the men. Miss Della Gleason, of this city, daughter of Dr. Gleason, has been attending medical lectures in this University, and has graduated with the highest honors.

---

INFINITESIMAL DOSING.—Dr. John C. Peters, in his address on "Sects in Medicine," says: "The Tartar physicians, or Llama doctors, have long superseded infinitesimal doses, as, if they do not happen to have any medicine with them, they are by no means disconcerted, for they merely write the name of the remedy they wish to give on a little scrap of paper, moisten this with saliva, roll it up into a pill, which the patient tosses down his throat with the same perfect confidence as he would aloes, assafœtida, or any other remedy. To swallow the name of the remedy or to take the medicine itself, say the Tartar physicians, by any patient comes to precisely the same thing. If paper is not at hand, the name of the drug is written with clay or chalk upon a board, which is then washed off, and the patient swallows the liquid."

"MANIFESTATIONS." — Another mountebank, named Warren, recently gave our citizens an exhibition of "spiritual manifestions," in Stancliff Hall. It proved to be a very low order of trickery, and how people can be entertained, much less mystified, by that species of jugglery, is beyond our ken. We can produce a gentleman in this city who can do all Mr. Warren's "manifestations," in a much better manner, and *very many more of them;* in fact, can astonish that individual and any of his believers, out of their very boots, and do it all without aid from the "spirits," or any other chap.

---

CHLOROFORM DEATH.—Dr. Freuzal reports, in the *Progres Medical,* a case in which a child, apparently dead from the effects of chloroform, was recalled to life by inversion and suspension by the feet, and forced movements of the chest. The face and lips were discolored, and there was neither heart action nor respiration.

This is the plan devised by the celebrated surgeon Nelaton, and is almost invariably successful. In addition to the suspension and movements of the chest, the tongue should be drawn from the mouth with a hook or tenaculum. We recently had a case in our own practice, in which a child was saved by this method.

---

A DIFFERENCE IN MEDICAL VIEWS.—In view of the following actual occurrence, it is at once seen that it is extremely hazardous for man and wife to hold discrepant medical views. A policeman reports that he noticed on his beat a house with two smashed windows. In attempting to ascertain the cause, he observed a man laying upon the floor, with a bleeding scalp, and an infuriated woman standing over him with a bloody poker. Inquiring the cause of the rumpus he was enlightened by the woman as follows: "You see that chap there? Well, he's my husband. Baby's sick—he said give her castor-ile;—I said give her goose-grease, he said no, you don't—and there he lays!"

---

CURIOSITY.—We have often observed, during our summer ramblings in the woods, the desire or intuition of nearly all animals and birds, to inspect any new or mysterious object that might attract their attention. We have frequently called birds to a tree, behind which we were concealed, by producing strange whistlings. Have attracted squirrrels and other small animals, by the wave of a handkerchief. Sportsmen frequently attract deer within easy rifle range, by concealing themselves in the grass, and waving a red handkerchief from the top of a gun. We find this same 'curiosity" revealed in the human race,—a strong, argument, perhaps, for the Darwinian theory—and have seen in a lecture-room, a large audience, seemingly much interested in a charming discourse from their pastor, where more than two-thirds of them would turn their heads toward the door, to see who it was came in last. One of two things must be done. Either our friend of the Bazar must cease coming late, or that latch upon that particular door must be packed in rubber, to prevent its rattle.

---

DISAGREEABLE.—Were we to have a call from a particularly disgusting and annoying individual, and desired to hurl an abusive and infamous epithet at him, we should call him *March.* It would indicate our desire with reference to his movements, and at the same time name him for one of the most disgusting, abominable, miserable months of the year. While we write, we have a pain in our head, an ache in our back, a shiver in our limbs, a buzzing in our ears, a stoppage in our nose, and and an infernal blustering out-doors. "Gentle Spring!" Bah,—gentle mother-in-law;—we had rather be with Greene on top the Rocky Mountains, or with McIntyre catching seals in Alaska. No more "gentle spring" for us, if you please.

---

THAT WINTER.—The one just passed, we refer to,—confound it! we do not want to see another like it. It not only made a great many people ill, but rendered us so also, in trying to make them well again, to say nothing of the four hundred dollars paid Barney for coal, to keep them warm.

But, as we write in the pleasant twilight, the robins are calling their mates to their favorite nesting boughs, the windows are down at the top, the fire out in the grate, and the soft maples are beginning to show their bright-red buds.

Soon, it will be warm again, and as we throw the fly upon our favorite mountain streams, and, one by one, place the beautiful silvery trout in our creel, we shall have forgotten the unpleasant scenes of the old, blustering winter, and rejoice that we are capable of enjoying the beautiful sunshine, the mountain air, and nature's lovliness, as revealed in forest and stream. After all, does not the strong contrast of winter with summer give us a stronger relish for the latter, and are we not better prepared to acknowledge the wisdom of the Great God who created both winter and summer for our better enjoyment? We think so. Yet, *confound that winter!*

GRATEFUL.—A short time since, a clerical gentleman of this city, entertained, as is his wont, a properly accredited poor man, from a neighboring city. Said indigent individual remained with our kind hearted minister, for several weeks; was fed, clothed, and ministered to, in many kindly ways, until it culminated in his borrowing ten dollars from the minister for the purpose of leaving the city. Our clerical friend had the utmost confindence in his protege—in short, would have backed him with his bottom dollar, until an item in the morning *Advertiser* opened his eyes to the fact, that he was "a victim of misplaced confidence." The item referred to apprised the minister of the fact, that his friend had contracted marriage with a young woman in a neighboring house, and had *employed another preacher to tie the knot*, and what was worse, as was afterward learned, paid that identical ten dollar note for the service.

---

PATENT TENT.—We have heard from our managerial friend in Philadelphia. H. met him there recently, and informs us that he intends joining us on the Lycoming in June, with a lightning proof tent. He describes it as being lined with Risdon's plastic slate roofing—to shut out the vivid lightning, and covered with Sol. Bowles' tubular, copper lightning rods, to conduct the infernal and mysterious fluid into the ground. By this arrangement, he expects to enjoy the pleasures of camping out, without being frightened half to death with one of Bodine's first-class thunder storms. H. has joined him in the arrangement, so that happiness and contentment will reign supreme, (minus thunder and lightning,) in camp, this season. We discover but one fault in the tent—plastic slate roofing won't keep out anything—not even rain. Substitute a hoodwink and you're all right.

---

SCHOOL HYGIENE.—The Board of Education of Eastern Middlesex, Mass., have adopted a rule "to prevent the attendance in public schools of any child residing in a family where there is, or has been, cases of measles, scarlet fever, or whooping-cough, until the physician in attendance on such cases of disease shall have furnished a certificate that in his opinion the period of danger from infection is past, and that he knows the infected premises have been thoroughly disinfected."

A similar rule has been adopted by the Board of Education in this city. Parents are thoughtlessly, if not criminally, negligent in sending their children to school during the prevalence of such diseases in their families. It should not be permitted until all the provisions in the above rule are carried out. The child is capable of communicating scarlet fever to others, weeks after it has seemingly fully recovered from the disease. As long as a flake of the dead skin falls from its body—no matter how small—just so long is there danger in communicating the disease.

Parents cannot be too careful, therefore, in keeping their little ones at home until all danger is past, for of the two diseases, small-pox and scarlet fever, the latter is by far the most terrible in its consequences.

---

OUR IMPROVEMENTS.—It will be at once seen that we have adopted a very fine quality of tinted paper, and that the BISTOURY appears in an entirely new dress. The type and materials are all new, purchased expressly by the *Advertiser* Association, our printers, for the use of the BISTOURY. It will also be seen that a first-class job of printing has been done for us, under the supervision of Mr. J. S. Allen, foreman in the *Advertiser* establishment, than whom, a better book or magazine printer does not exist anywhere. These improvements have been brought about by reason of our much enlarged subscription list, our patrons kindly affording us the money necessary to carry them out. Great care will be taken to arrange the BISTOURY for binding, and at the end of three years, a copious index will be published, as in the January number. Preserve, therefore, your journals, and when bound, you will have the most valuable book for reference in disease or domestic wants, anywhere published.

---

A SUCCESS.—The Orphans' Home Fair, the Home Theatricals and the Martha Washington Tea Party, were each and severally grand and complete affairs. There were not quite "millions in it," but certainly thousands of dollars were collected in aid of Elmira's favorite charity, "The Southern Tier Orphans' Home." Our wives and sweethearts turned out handsomely—as they always do—and worked long and ardently for the success which they achieved. Of course, it is understood that the pronoun *our*, is not used editorially in this instance, but generally. We believe that all our pretty girls have beaux, hence are they sweethearts, and as *all* our girls are pretty, why, of course, they at once *all* become sweethearts—nothing plainer than that. And as our wives were once pretty girls, and "us fellows" (*us* editorially included, this time,) were splendid looking boys, why, it is plain to be seen that—see here, this is getting mixed;—what we started out to say was that, the various entertainments given in aid of the Orphans' Home were successful in the extreme, and having said it, will stick to it, but—quit.

---

"THERE'S MILLIONS IN IT."—As an evidence of the gullibility of the people, particularly when sick, and of the beneficial effects of advertising, we refer our readers to the subjoined list of sales of patent medicines, in one year, as published in the *Chemists' & Druggists' Circular*. The sums expended for nostrums is perfectly surprising, and is a sad commentary upon the influence exerted by the regular medical man upon community. It exhibits the fact that there are a surprising number of people who have more confidence in quack medicines than in regular physicians.—But look at the list of sales, and if you would reap an abundant harvest of riches, without caring *how*, just embark in the patent medicine swindle, advertise it liberally, and your fortune is made:

| | |
|---|---|
| Hostetter's Bitters | $1,000,000 |
| Drake's Bitters | 700,000 |
| Hoofland's Bitters | 100,000 |
| Ayers' Pills | 200,000 |
| " Pectoral | 150,000 |
| " Ague Cure | 100,000 |
| " Sarsaparilla | 150,000 |
| Mrs. Winslow's Soothing Syrup | 500,000 |
| Brown's Troches | 250,000 |
| Helmbold's Buchu | 500,000 |
| Kennedy's Discovery | 100,000 |
| Brandreth's Pills | 150,000 |
| Herrick's Pills | 100,000 |
| Schenck's Pills | 100,000 |
| Wright's Pills | 150,000 |
| Jayne's Expectorant | 100,000 |
| Sozodont | 100,000 |
| Hall's Hair Renewer | 400,000 |

---

MEDICAL ADVERTISING.—Formerly the regular profession were decidedly averse to *any* form of medical advertising. According to the Code, that great bug-bear to the timid professional, the physician is not permitted to have his card appear, nor allow his name to be seen in any newspaper, in connection with any surgical operation, or any matter pertaining to medicine or surgery. But now things have changed. We find the first medical journals of the land discussing such questions as "What is the limit of Newspaper Advertising?" "Should not the Regular Physician advertise his calling in the Public Prints?" &c., &c., which would seem to indicate a decided change in sentiment. Then, we find that most medical societies are fond of publishing their proceedings in the Daily Newspapers, ostensibly to preserve them in printed form, but *really* to advertise the members thereof to the public as capable men in that department of medicine of which their essays treat. We also observe that many of the medical professors in New York city are even going further and actually publishing their medical papers entire in the secular journals of that city.

For years we have advocated a modest system of advertising for the specialist, in order that people needing their services, might know where they could be found, and so prevent many from falling into the hands of traveling charlatans. We are glad to note the profession are waking up upon this matter and are attempting to prescribe what shall be considered orthodox medical advertising. We are anxious to see what the final prescription will be.

